# Management of anastomotic leak following restorative rectal cancer resection—a multi-centre retrospective audit in Ireland

**DOI:** 10.1007/s11845-026-04335-w

**Published:** 2026-04-22

**Authors:** R. Walsh, B. Creavin, E. Murphy, T. K. Daly, K. V. Subramanian, N. D. Kennedy, L. Byrne, M. Flanagan, É. J. Ryan, J. P. Burke, M. Joyce, P. H. McCormick, J. J. Mulsow, R. A. Cahill

**Affiliations:** 1 UCD Centre for Precision Surgery, School of Medicine, University College Dublin, Dublin, Ireland; 2 Department of Colorectal Surgery, St James’s Hospital, Dublin, Ireland; 3 Department of Colorectal Surgery, Beaumont Hospital, Dublin, Ireland; 4 Department of Colorectal Surgery, Galway University Hospital, Galway, Ireland; 5 Department of Colorectal Surgery, Mater Misericordiae University Hospital, Dublin, Ireland

**Keywords:** Anastomotic Leak, Conservative Management, Drainage Procedures, Management Strategies, Pelvic Sepsis, Rectal Cancer

## Abstract

**Background:**

Anastomotic leak (AL) following rectal cancer resection significantly impacts outcomes. Currently its management management is not standardised. This study reviews AL management following restorative rectal cancer resection in Ireland.

**Method:**

A retrospective review of centres’ own databases and HIPE-coded data between 1st January 2020-31st December 2023 was performed to identify AL patients and their characteristics and interventions. The primary outcome of “success” in management was defined as the resolution of pelvic sepsis and/or healing of the anastomotic defect. Secondary outcomes included long-term stoma rates, length of stay, 30-day mortality dates, major morbidity rates, disease-free and overall survival.

**Results:**

36 patients were identified across four centres with the median follow up being 26.9 months. Nine (25.0%) patients received conservative management (CM), fifteen (41.7%) were treated with drainage procedures (DP)(66.7% percutaneous radiological drains, 33.3% transanal drains) and twelve had surgery (4, 11.1%, had anastomosis conserving surgery-ACS- and 8, 22.2%, anastomotic takedown-SAT). The success rate of these interventions was CM:77.7%, DP:73.3%, ACS:100.0%, and SAT:75.0%. Long-term stoma rates were CM:20.0%, DP:46.7%, ACS:50.0%, and SAT:87.5%. Length of stay was CM:18 days (IQR 12–21), DP:23 days (14–30), ACS:26 days (19–35), and SAT: 27 days (7–45). There was no 30-day mortality. There was no significant difference in disease-free and overall survival by management (p = 0.509, p = 0.395 respectively).

**Conclusion:**

Management for AL following rectal cancer resection varies but is generally successful. While patient factors and institutional expertise influences management opportunities and strategies, some investment, including in data collection and analysis, could better standardise care in and between centres.

## Introduction

Anastomotic leak (AL) is one of the most feared complications after rectal cancer resection. As well as resulting in increased morbidity, mortality and length of hospital stay, AL has negative impacts on oncologic and functional outcomes [1–4]. It is reported to occur in 5–25% of patients following rectal cancer resection [5–9], an up to four-fold increase compared to colonic resections [10], with incidence influenced by patient characteristics as well as disease and surgical factors. Although there is an increasingly robust evidence base for AL reduction strategies, including the use of mechanical and oral antibiotic bowel preparation, defunctioning loop ileostomy creation, and indocyanine-green angiography [11–17], the evidence base for AL management is limited.

Historically, AL was managed with radical surgical intervention with takedown of the anastomosis resulting in an end stoma. There is now a shift towards less invasive strategies including interventional radiology-guided percutaneous drainage, endoscopic vacuum therapies, and conservative management. However, the evidence base for each is largely single-centre series focused on a single intervention type with large heterogeneity in outcome reporting limiting the ability to draw comparisons between different approaches [18]. While there are professional guidelines on the prevention and management of anastomotic leak based on expert opinion, given the lack of robust evidence in this area, these guidelines lack specific indications or protocols to direct patients to the most appropriate management pathway [19, 20]. Thus, surgeons are left to rely on experience, preference, and resource availability.

Within the Republic of Ireland, rectal cancer surgery is centralised to a number of National Cancer Centres as part of a broader effort to optimise oncologic outcomes and streamline perioperative care [21]. This strategy has improved patient survival significantly [22] and provides the opportunity for ongoing evolution of surgical care via the Rectal Cancer Surgical Leads group and participating surgical teams and centres. The aim of this study was to describe the approach to the management of AL in these centres with the associated short- and long-term outcomes.

## Methods

Invitation to collaborate in this retrospective study was extended via rectal cancer surgical leads in each National Cancer Centre in Ireland. Seven centres agreed to participate with two being unable to due to resource and time constraints.

With audit committee approval in each participating centre, patients with AL were identified via local (prospectively maintained cancer databases, morbidity and mortality meeting databases) and Hospital Inpatient Enquiry (HIPE) coded database searches. In brief, centres looked to identify patients over the age of 18 with AL diagnosed between January 2020 and December 2023 following an elective, restorative anterior resection for histologically proven rectal (i.e. those within 15 cm of the anal verge) adenocarcinoma. AL was defined as a defect in the bowel wall at the site of surgical anastomosis leading to communication between the intra- and extra-luminal compartments. Acute AL was defined as occurring within 30 post operative days with late AL being those occurring after 30 days. Rectal tumours were subcategorised as low (≤ 5 cm from anal verge), middle (5–10 cm from anal verge), and high (≥ 10 cm from anal verge). Patients were excluded if they had a non-restorative anterior resection, abdominoperineal resection, non-malignant pathology, or a diagnosis of AL outside of the study period. Patient demographics, disease factors, and details of index surgery were synthesised into a tabulated summary.

Conservative management (CM) of AL was defined as antimicrobials, nil per os, total parenteral nutrition, and/or other supportive care. Drainage procedures (DP) were defined as any drain placed post-operatively intended to resolve the collection from AL. These were categorised into transanal drains (placed via examination under anaesthesia (EUA) ± flexible sigmoidoscopy), and percutaneous drains (placed in interventional radiology (IR)). Transanal drains could be passive or active (e.g. Endo-SPONGE® endoluminal vacuum therapy). Percutaneous drains were pigtail catheters on passive drainage placed with CT, ultrasound or fluoroscopic guidance in a dedicated interventional radiology suite, which were upsized, replaced, or repositioned if needed based on drain output, patient status, or the size of the collection on subsequent imaging. Repeat procedures for replacement or upsizing of drains were recorded. Anastomosis conserving surgery (ACS) was defined as any laparoscopic or open procedures to address AL which preserved the original anastomosis, this included the creation of a defunctioning stoma (when one was not created before or during the index operation), washout, oversewing of the defect, and surgical drainage. Surgery with anastomotic takedown (SAT) was defined as a laparotomy with takedown of the anastomosis and formation of an end stoma.

Outcome measures were chosen after review of relevant literature [23]. Short-term measures for each management strategy were the success rate, 30-day mortality rate, length of stay (LOS) and repeat intervention rate associated with the chosen intervention. Long-term measures were the long-term stoma rate, disease-free and overall survival. Success was defined as resolution of pelvic sepsis and/or healing of the anastomotic defect. Repeat intervention for drainage procedures was defined as the instances of replacement, repositioning, or upsizing of the given drain. Long-term stoma was defined as either a loop or end, colostomy or ileostomy at the end of the follow-up or until death.

Statistical analysis was conducted using SPSS statistics (v.21.0; IBM, Armonk, New York, USA). Descriptive statistics were used to report on patient and disease characteristics. Continuous data are presented as medians (inter-quartile range) or means (standard deviation) if normally distributed. Overall and disease-free survival were estimated using Kaplan–Meier curves and differences were investigated using a log-rank test. Results were considered to be statistically significant when p < 0.05.

## Results

**Fig. 1:**
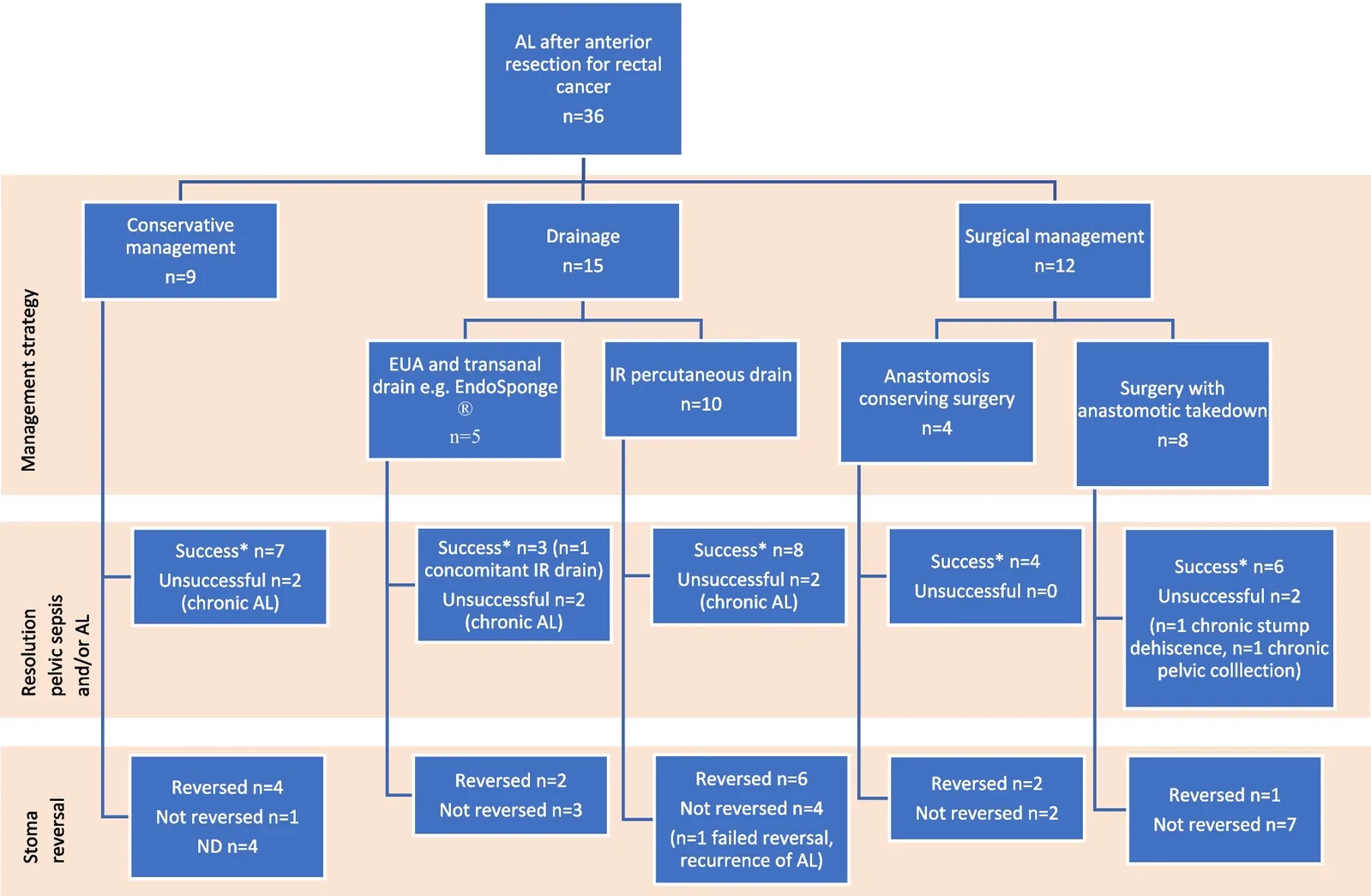
Flow diagram of the management of the AL cohort, with success and stoma reversal data

**Fig. 2:**
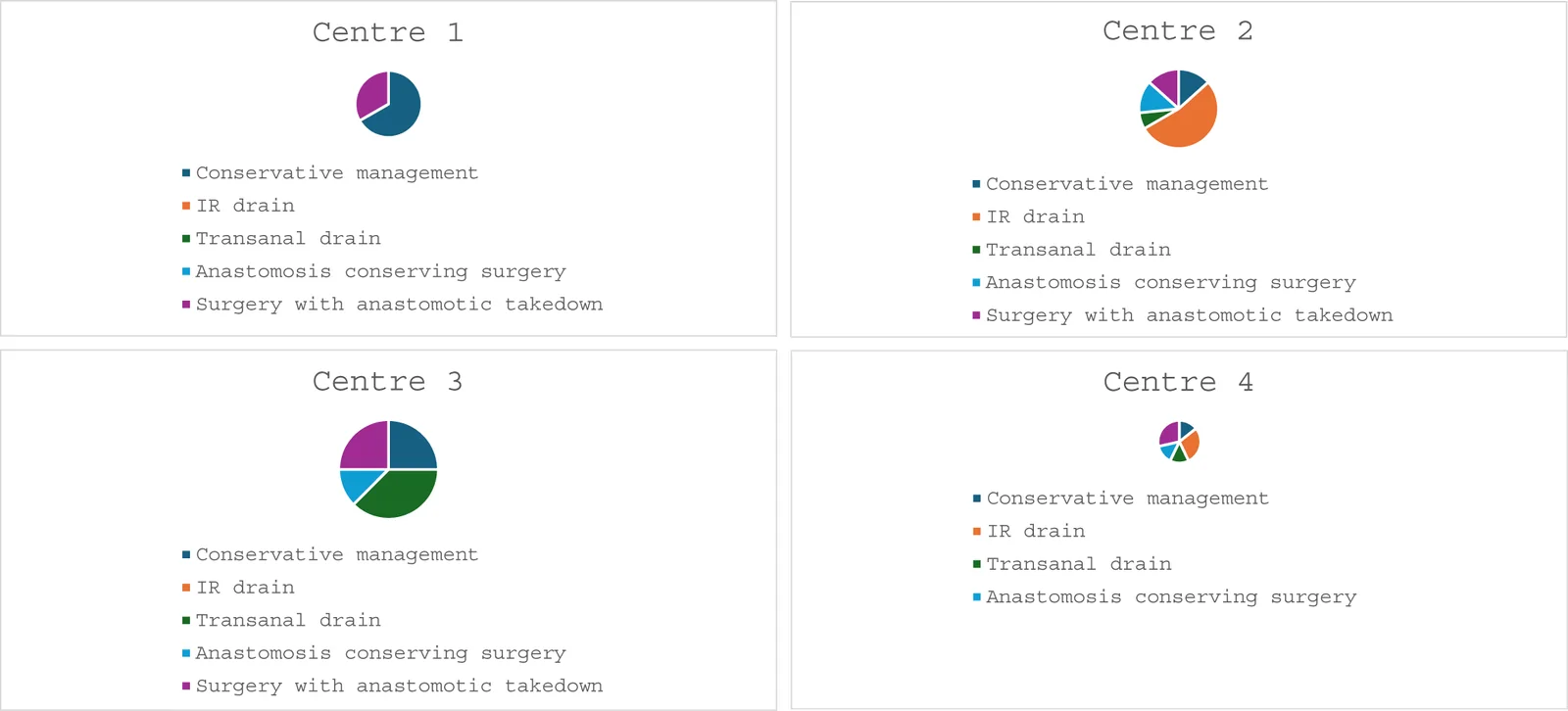
Management strategies utilised in each centre

Four NCCP centres contributed complete data within the six month data collection window with three being unable to due their constrained resource availability. There were 36 patients with anastomotic leak reported, diagnosed by radiological or endoscopic investigation. A flow diagram of the pathways for these patients is shown in Fig. 1. The median follow up was 26.9 (IQR 18.7–44.4) months. The utilisation of the various management strategies varied between centres, as seen in Fig. 2.

### Patient demographics

Patient demographics divided by management cohort are detailed in Table 1. Of note, the mean age and BMI was similar across cohorts. The male prevalence was notably lower in the SAT cohort (62.5%, compared to > 75% for CM, DP, and ACS). Over 2/3 of patients in the CM, DP, and ACS cohorts had an American Society of Anaesthesiologists (ASA) score of 1 or 2, whereas only half of the SAT cohort had an ASA score of 1 or 2. In terms of tumour height, approximately one third of the CM and SAT cohorts (3/9, 2/7) had low rectal tumours, compared to half of the DP and ACS cohort (7/14, 2/4). All patients in the CM and DP cohorts had defunctioning ileostomies created before or during the index procedure, compared to half of the ACS cohort (2/4) and approximately two thirds of the SAT cohort (5/8).

**Table 1:** Patient demographics and disease characteristics in each cohort

	Conservative management N = 9	EUA and transanal drainage (e.g. Endo-SPONGE®) N = 5	Percutaneous IR drain N = 10	Anastomosis conserving surgery N = 4	Surgery with anastomotic takedown N = 8
Age	61.9 (± 5.5)	68.8 (± 9.3)	57.8 (± 10.1)	63.0 (± 16.2)	57.6 (± 9.2)
Male:Female ratio	8:1	5:0	3:2	3:1	5:3
ASA I	0	0	0	0	0
II	6	3	9	3	4
III	3	2	1	1	4
IV	0	0	0	0	0
CCI score	4.4 (± 1.2)	4.8 (± 1.5)	3.7 (± 1.0)	5.0 (± 2.7)	4.3 (± 1.1)
Active smoker	11.1%	20.0%	0%	25.0%	12.5%
BMI	27.5 (± 4.2)	30.3 (± 6.8)	26.8 (± 3.8)	29.5 (± 4.5)	27.5 (± 2.7)
Diabetes mellitus	0%	0%	20.0%	25%	12.5%
Immunosuppression	0%	0%	0%	0%	0%
Neoadjuvant therapy	77.8%	60.0%	90.0%	50%	50%
Height of tumour*:					
High	2	1	2	1	3
Middle	4	1	3	1	2
Low	3	3	4	2	2
Missing	0	0	1	0	1
Operation:					
Laparoscopic	4	3	6	4	5
Robotic	0	1	0	0	0
Open	2	0	3	0	1
Converted	3	1	1	0	2
Defunctioning ileostomy	100%	100%	100%	50%	62.5%
Intraoperative drain	88.9%	83.3%	55.5%	75%	62.5%
Indocyanine green use	11.1%	60%	0%	0%	12.5%
Length of operation (min)	327 (± 102)	320 (± 63)	292 (± 72)	309 (± 36)	262 (± 91)
Pathological T stage:					
ypT0	0	0	1	0	1
ypT1	1	0	1	0	1
ypT2	2	4	5	1	3
ypT3	5	1	1	3	3
ypT4	1	0	2	0	0
Pathological N stage:					
ypN0	5	3	8	2	5
ypN1	3	2	2	1	3
ypN2	1	0	0	1	0
R status:					
R0	8	5	10	4	8
R1	1	0	0	0	0

Presented as absolute figures, mean (± standard deviation), or percentages. *Rectal tumour height was categorised as low (≤ 5 cm from anal verge), middle (5-10cm from anal verge), and high (≥ 10 cm from anal verge)

EUA; examination under anaesthesia, IR; interventional radiology, ASA; American Society of Anaesthesiologists, CCI; Charlson Comorbidity Index, BMI; Body Mass Index

### Short- and long-term outcome measures

**Fig. 3:**
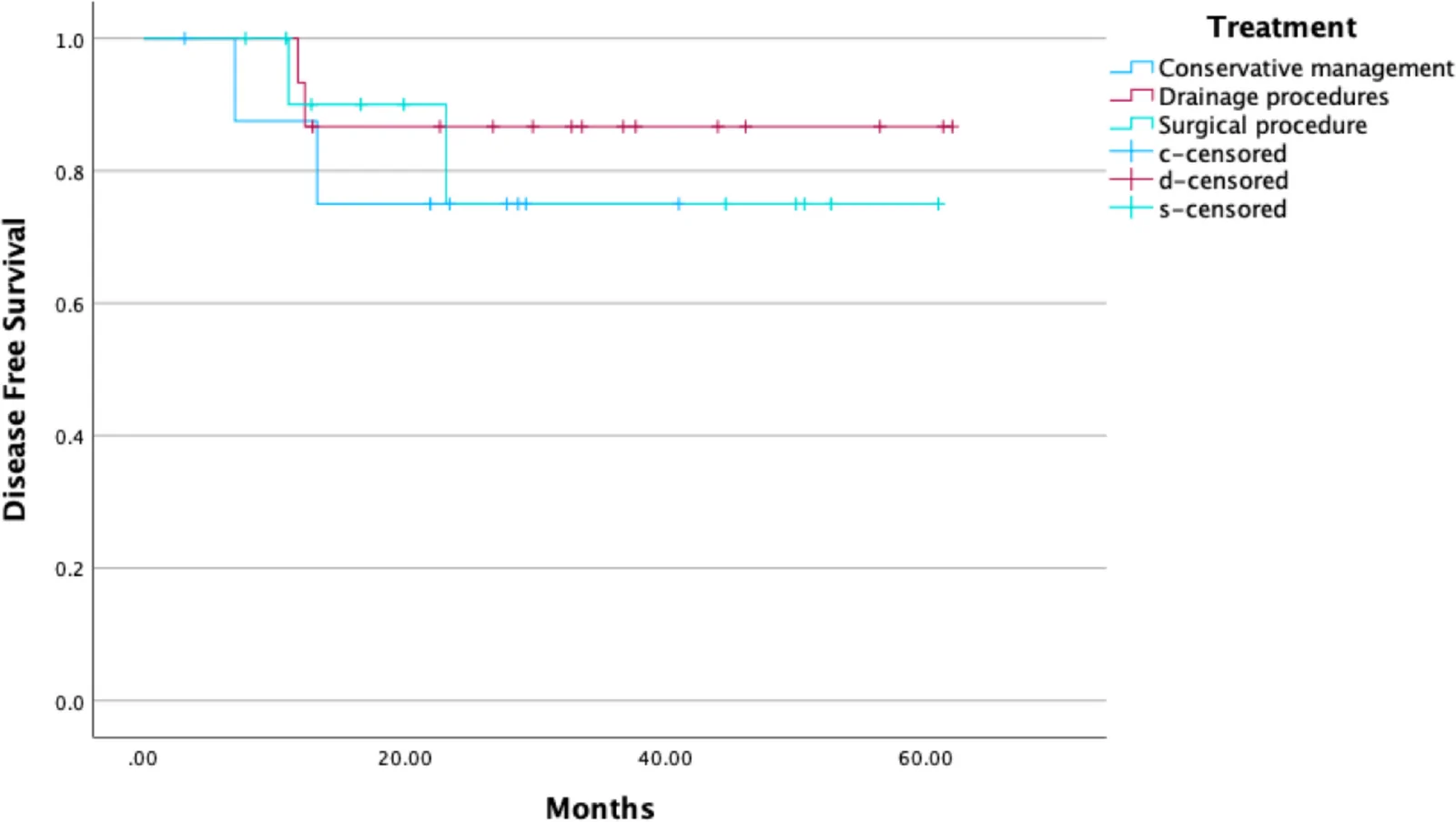
Disease-Free Survival (DFS) in the categories of conservative management, drainage procedures, and surgical procedures

**Fig. 4:**
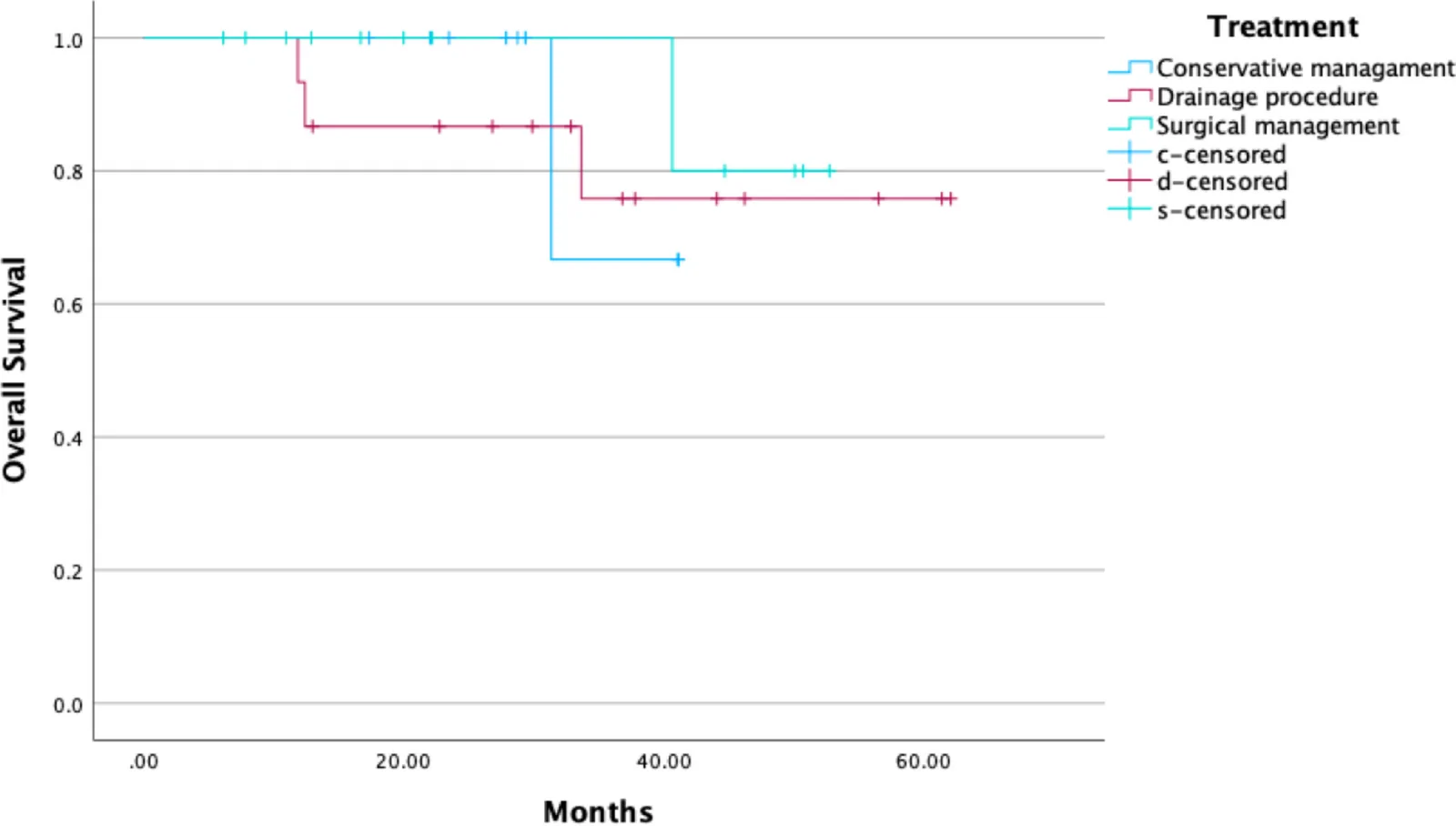
Overall survival (OS) in the categories of conservative management, drainage procedures, and surgical procedures

There was no 30-day mortality. Details of the morbidity in each management cohort are shown in Table 2. Kaplan–Meier curves for the disease-free and overall survival are shown in Fig. 3 and 4 respectively. There was no significant difference in disease-free and overall survival between management cohorts (p = 0.509, p = 0.395 respectively).

**Table 2:** Inpatient morbidity in each management cohort from time of index operation

	Conservative management N = 9	Transanal drainage (e.g. Endo-SPONGE®) N = 5	Percutaneous IR drain N = 10	Anastomosis conserving surgery N = 4	Surgery with anastomotic takedown N = 8
Respiratory	1	0	3	0	0
Cardiac	1	0	1	0	0
Renal	0	0	0	0	0
VTE	0	0	0	1	0
GI	2	1	1	0	3
Bleeding	0	0	1	0	1

Respiratory complications included pneumonia, effusion, atelectasis. Cardiac complications included atrial fibrillation, heart failure. Renal complications included acute kidney injury. VTE; venous thromboembolism, included pulmonary embolism and deep vein thrombosis. GI; gastrointestinal complications included ileus, high output stoma, obstruction. Bleeding complications included any intra- or post operative intraabdominal haemorrhage. Urological complications included urinary tract infections or urological injuries. SSI; surgical site infection

Conservative management: Nine of the 36 patients (25.0%) had CM. The median length of stay (LOS) was 18 days (IQR 12–21). Median follow up for this cohort was 28 months (IQR 20–35). Success rate was 7/9 (77.7%) with the remaining two patients having a chronic leak on follow-up. Long-term stoma status was recorded in 5/9 patients, 1/5 (20.0%) noted to have a long-term stoma.

Drainage procedures: Fifteen of the 36 patients (41.7%) had DP (10/15 (66.7%) percutaneous IR drains, 5/15 (33.3%) EUA and transanal drains). Overall success rate was 11/15 (73.3%). The remaining four patients had a chronic leak on follow-up investigation. The success rate of EUA and transanal drain was 3/5 (60.0%), and 8/10 (80.0%) for IR percutaneous drain. Median length of stay for all DP was 23 days (IQR 14–30). Median LOS for IR percutaneous drainage was 17 (IQR 13–27), and 31 (IQR 22–45) for EUA and transanal drain. The median repeat interventions was 6 (IQR 4–9) for EUA and transanal drainage. Only 1/10 patients with IR percutaneous drains had a repeat intervention on one occasion, with the remaining undergoing no repeat interventions. Median follow up for this cohort was 34 months (IQR 23–46). In terms of long-term stoma rate, DP was 7/15 (46.7%) overall, with 3/5 (60.0%) for EUA and transanal drain and 4/10 (40.0%) for IR percutaneous drain.

Surgical management: Four of the 36 patients (11.1%) had ACS, and 8 (22.2%) had SAT. The success rate of surgical management was 4/4 (100.0%) for ACS and 6/8 (75.0%) for SAT, with the remaining two patients having a chronic stump dehiscence (n = 1) and chronic pelvic collection (n = 1). Median length of stay was 26 days (IQR19-35) for ACS, and 27 days (IQR 7–45) for SAT. Median follow up for this cohort was 31 months (IQR 15–50). The long-term stoma rate for ACS was 2/4 (50.0%), and SAT was 7/8 (87.5%). The management utilised in the four patients with ACS was laparoscopy and washout, laparotomy and washout, laparoscopic defuntioning ileostomy formation and washout, and laparoscopic defunctioning colostomy formation and washout.

### Late AL

Six of the 36 patients had late AL. Table 3 details these cases. 1/6 (16.7%) underwent conservative management which was unsuccessful with chronic AL and stoma. 2/6 (33.3%) had EUA and transanal drainage, one being unsuccessful with chronic AL and the other being successful regarding sepsis resolution but both patients have a long-term stoma in situ. 1/6 (16.7%) had an IR percutaneous drain which was successful regarding sepsis resolution but had a long-term stoma in situ. Finally 2/6 (33.3%) had AL post reversal of defunctioning ileostomy which was managed with surgery with anastomotic takedown, both having resolution of sepsis and one being able afterwards to have restoration of bowel continuity.

**Table 3:** Description of the 6 cases of late anastomotic leak

	Time of diagnosis	Method of diagnosis	Management strategy	Success	Long-term stoma
P1	3 months	Surveillance CT TAP	Conservative – watch and wait	N—chronic pre-sacral collection	Y
P2	9 months	Surveillance CT TAP	EUA and transanal drain	Y	Y
P3	2 months	Surveillance CT TAP	Percutaneous IR drain	Y	Y
P4	54 months	CTAP post reversal of defunctioning ileostomy	Surgery with anastomotic takedown	Y	Y
P5	13 months	CTAP post reversal of defunctioning ileostomy	Surgery with anastomotic takedown	Y	N
P6	33 days	GGE pre-reversal of defunctioning ileostomy	EUA and transanal drain	N – chronic anastomotic defect	Y

CT TAP; computed tomography thorax abdomen pelvis, CTAP; computed tomography abdomen pelvis, EUA; examination under anaesthesia, IR; interventional radiology, GGE; gastrograffin enema

## Discussion

This multi-centre retrospective audit across four national rectal cancer centres in Ireland provides insights into the contemporary management of AL following restorative rectal cancer resection in this country. Although this study focusses on AL management, crudely, it indicates the approximate incidence of AL in such patients to be 4.5–6% (based on NCCP figures of 350–400 patients per year having rectal resection for cancer with 21% of these being non-restorative). Across the country this estimates three patients suffer AL during any two month period with there being, on crude average, 2–3 AL per centre per year. This however is likely to be an underestimate of the true incidence as it seems unlikely every leak was captured by our study method and some patients presenting late or elsewhere or those having subclinical AL are likely to have been under counted. The crude rate fits at the lower side of the range reported in the literature although notably other centre and country (such as the UK and the Netherlands) experience with quality improvement initiatives and formal controlled studies often start too with number in this range that rises then to around 20% when more careful and complete examination for AL is undertaken and the study period and inclusion criteria broaden [5, 24].

This study reflects real-world clinical management decision-making in the absence of definitive guidelines, highlighting key differences in the utilisation and outcomes of various strategies. There is increasing international effort to understand AL better and reduce the impact this complication has on patients and the health service, most especially on measures to reduce its incidence [11–17]. However, there has been as yet less effort expended on standardising AL management strategies in and between clinical units generally. While the overall success rates of the four intervention types reported here were relatively high, each approach demonstrated distinct strengths and limitations in terms of hospital stay, stoma outcomes, and repeat intervention burden (although the nature of this retrospective study means we could not elicit the underlying reasoning for specific intervention deployment). Nonetheless high success rates reported across all treatment groups are encouraging and support an individualised approach to AL management [19, 20]. Patients who underwent conservative management showed mostly favourable outcomes in terms of hospital length of stay and long-term stoma formation, suggesting that non-interventional strategies can be utilised [19, 20]. All patients in this cohort had primary defunctioning ileostomies, raising the question of whether the defunctioning ileostomy lowered the initial clinical severity of the leak leading to less invasive management or whether healing was favoured with faecal diversion. However, this cohort was small and likely included patients with less severe leaks and favourable clinical status.

Drainage procedures, representing the most common intervention strategy in this cohort, were split between transanal and IR-guided percutaneous approaches. This cohort also was utilised exclusively in those with primary defunctioning ileostomies. IR-guided drainage seems to demonstrate better outcomes in terms of success, fewer repeat interventions, and shorter hospitalisation. Transanal drainage incurred a high repeat procedural burden by comparison, as is expected with this management pathway. These findings suggest that while transanal drainage remains a viable option, particularly where percutaneous access is anatomically unfeasible, the high resource utilisation and patient burden should be weighed carefully [25]. These variations in outcomes may also reflect the initial presentation as perhaps large defects with more severe leakage were selected for transanal drainage while contained leaks with a small defect were those amenable to IR-guided drainage. The variation in outcomes between drainage modalities underscores the need for prospective comparative studies and cost-effectiveness analyses to guide optimal resource allocation. Surgical options including ACS and SAT were employed in patients with lower rates of primary defunctioning ileostomies and higher ASA scores. Whilst the high success rate seen with ACS is notable, the high long-term stoma rate raises questions about functional outcomes and eventual stoma reversal success. SAT had the highest long-term stoma rate, a predictable outcome given the inherent nature of the operation. Although SAT remains a definitive option, particularly in patients with intra-abdominal sepsis or failed prior interventions, it carries significant long-term implications on quality of life [20, 26–28].

The six cases of late AL provide further nuance to the findings. Late AL presents a distinct clinical challenge and also lacks clear consensus on management timing and modality [29]. The variation in treatment strategies employed in this subgroup indicates that late AL can present with a spectrum of clinical severity and is not inherently more amenable to any single treatment strategy. It is also notable that management of these late AL cases resulted in a high failure rates and long-term stoma rate. This correlation between delayed presentation and poor functional recovery should be prioritised in future research, perhaps investigating the role of screening for AL to avoid these late presentations. Importantly, this study found no significant difference in disease-free or overall survival across the management cohorts. However, the long-term quality of life, particularly in patients with permanent stomas or chronic pelvic sepsis was not recorded. In this regard, future cohorts including patient-reported outcomes would provide additional context to these management strategies.

Overall the heterogeneity of management techniques is a key finding of this study. The lack of definitive consensus on appropriate management likely influences the variation seen with local factors (including surgeon and institutional preference and experience as well as resource availability and patient factors) impacting care. The variation in outcomes supports the call for national and international guidelines that incorporate stratified pathways based on leak severity, patient fitness, and anatomical considerations. Whilst several algorithms have been proposed, there remains minimal high-level evidence [19, 20, 30]. Furthermore, the current ISREC grading system for AL categorises severity based on the definitive management used rather than physiological, biochemical, endoscopic and radiological parameters [31]. The lack of a classification system incorporating these factors thus limits the comparison of management strategies due to the wide variation in clinical presentations. Generation of a standardised severity scoring system incorporating these factors would help develop more robust guidelines and care algorithms. In addition, prospective registries and national audits utilising a Core Outcome Set (c.f. COMET Initiative, Study 3343) and inclusion of quality-of-life metrics would help refine and validate optimal AL care pathways.

As a retrospective analysis, the study is limited by inherent selection and information biases and by being confined to one geographic jurisdiction. In addition, only four of nine centres submitted complete data indicating a need to better resource data collection and access regarding surgical outcomes generally. Clinical severity at the time of AL diagnosis, an important determinant of management strategy, was not recorded, limiting the ability to adjust for case complexity. Furthermore, as stated above, the figures here likely represent an underestimate of the true incidence of AL. Additionally, the small sample size reduces the generalisability of subgroup outcomes. This also impacted our ability to determine relative importance of risk factors and predictors including for instance by multivariate analysis with useful statistical power. Furthermore, the small numbers overall, per centre and indeed for any individual surgical technique and the limited time period (making it really a ‘snapshot’ audit) precluded by design calculation of more granular AL incidence as it would be unreliable to draw conclusions from this work. This was discussed a priori between the centres and the NCCP surgical rectal cancer lead group and agreed outside the scope of the study scope and also felt important to maximise participation. Despite these limitations, the strength of the study lies in its multi-centre nature, representation of real-world clinical practice and comparison across the full spectrum of management options. It also brings clarity to issues needing address in any further prospective work including how to better and more easily capture data, how to reduce incidence and improve care and greater crystalisation of individualised patient care protocols (including indeed defining reliable criteria for conservative care candidacy).

## Data Availability

Additional data have not been published in a public repository. The authors agree to make the data, analytic methods and study materials available to other researchers. These can be obtained by contacting the corresponding author using the details provided.
